# Satellite Observations of Atmospheric Ammonia Inequalities Associated with Industrialized Swine Facilities in Eastern North Carolina

**DOI:** 10.1021/acs.est.4c11922

**Published:** 2025-01-29

**Authors:** Akirah Epps, Isabella M. Dressel, Xuehui Guo, Maghogho Odanibe, Kimberly P. Fields, Ann Marie G. Carlton, Kang Sun, Sally E. Pusede

**Affiliations:** † Department of Environmental Sciences, University of Virginia, Charlottesville, Virginia 22904, United States; ‡ Carter G. Woodson Institute for African American and African Studies, University of Virginia, Charlottesville, Virginia 22904, United States; § Department of Chemistry, University of California Irvine, Irvine, California 92697, United States; ∥ Department of Civil, Structural and Environmental Engineering, University at Buffalo, Buffalo, New York 14260, United States; ⊥ Research and Education in eNergy, Environment and Water (RENEW) Institute, University at Buffalo, Buffalo, New York 14260, United States

**Keywords:** ammonia, environmental racism, concentrated animal feeding operations (CAFOs), satellite observations, infrared atmospheric sounding interferometer (IASI)

## Abstract

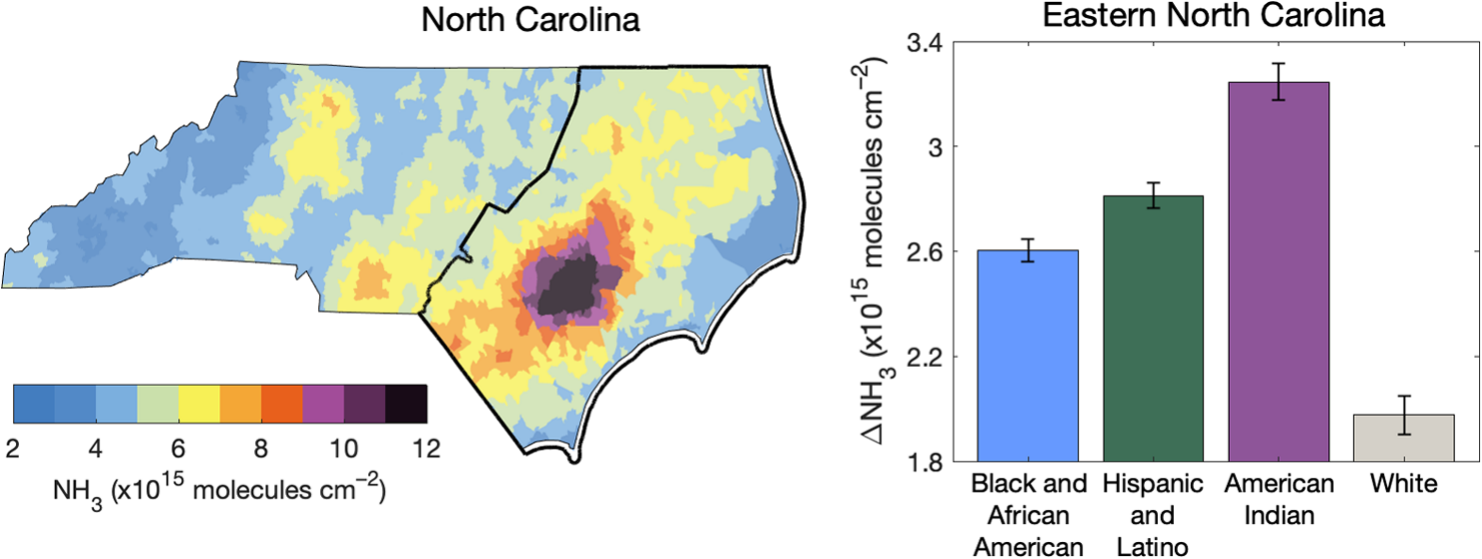

Industrialized swine facilities adversely affect the health and well-being of Eastern North Carolina residents in the U.S. and are an issue of environmental racism. Concentrated animal feeding operations (CAFOs) emit various harmful and noxious air pollutants, including ammonia (NH_3_). There are limited measurements of CAFO-related air quality, contributing to disputes around its severity. We use NH_3_ vertical column densities from the space-based Infrared Atmospheric Sounding Interferometer (IASI) to report systematic, distributive inequalities in NH_3_ column enhancements (ΔNH_3_ columns), equal to NH_3_ columns less an observationally determined tropospheric background. Population-weighted block group-scale ΔNH_3_ columns are higher by 27 ± 3% for Black and African Americans, 35 ± 3% for Hispanics and Latinos, and 49 ± 3% for American Indians compared to non-Hispanic/Latino whites in Eastern North Carolina (April–August 2016–2021). Surface winds and air temperature influence block group-scale NH_3_ distributions, with higher absolute NH_3_ inequalities for all groups on calm days and for Black and African Americans and Hispanics and Latinos on hot days, consistent with effects from NH_3_ volatization downfield of facilities from, e.g., manure-covered fields, particles, and other surfaces. ΔNH_3_ columns correspond spatially with permitted swine facilities, with residents living multiple kilometers from swine CAFOs chronically exposed to elevated NH_3_. Trends in NH_3_ columns over 2008–2023 are driven by regional-scale atmospheric processes rather than localized NH_3_ changes in CAFO emissions. Results are discussed in local decision-making contexts that have broad relevance for air quality issues without protective federal regulatory standards.

## Abstract 2

Observational evidence of distributive ammonia inequalities associated with swine CAFOs in Eastern North Carolina measured from space with IASI.

## Introduction

Concentrated animal feeding operations (CAFOs) are sources of air and water pollution and other nuisances in Eastern North Carolina in the U.S.^1−3^ Residents report noxious and nauseating odors, health and mood issues, and the violence of being misted with swine feces and urine by manure irrigation practices.^2,4−8^ Analyses of proximity to permitted facilities and atmospheric model simulations have demonstrated that CAFOs producing swine, other animals, and the related air pollutants are disproportionately located near the homes and schools of Black and African Americans, Hispanics and Latinos, and American Indians.^1,9−12^ However, systematic distributive air pollution inequalities associated with CAFOs have not yet been shown observationally. Satellite measurements are spatially comprehensive and collected independently of state and local governments and influential industries, providing empirical evidence of air quality impacts in locations where there are no air monitors and where residents’ claims are contested, supporting accountability around exposure to CAFO-related air emissions.

CAFOs are industrial livestock facilities that confine over 1000 animal units (based on pounds of live weight), typically swine, dairy cows, beef cattle, or chickens. CAFOs are sources of numerous air pollutants, including ammonia (NH_3_), hydrogen sulfide (H_2_S), methane (CH_4_), volatile and semivolatile organic compounds, bioaerosols, and biological contaminants and allergens.^3,13−18^ These pollutants affect ecosystems, climate, and human health, and their complex mixtures potentially have additional interactive exposure impacts.^19−22^ CAFOs are characterized by strong odors that are life-altering for nearby residents.^2^ In Eastern North Carolina, residents have described odors that are overpowering, causing gagging, nausea, and vomiting, permeating homes and clothing, forcing windows to remain closed, and limiting outdoor activity.^3,8^ People living close to CAFOs with swine have reported lowered immune function, higher levels of stress, anxiety, depression, and fatigue, and more frequent headaches, sore throats, runny noses, coughing, and irritated eyes, symptoms consistent with occupational exposures in swine confinement buildings.^2,4−7,9,13^ CAFOs can also affect residents financially. Across the U.S. and in North Carolina, CAFOs are associated with decreases in residential property values and regional economic growth rates because of reduced purchases in local stores and disruptions to community social and economic systems compared to farms and/or smaller operations.^23−29^

CAFO-related air quality is largely unregulated in the U.S. The location of the subset of CAFOs that discharge into navigable waters is public record because they are permitted under the U.S. Clean Water Act through the National Pollutant Discharge Elimination System (NPDES).^30^ North Carolina is the third largest pork producing state in the U.S.^31^ In North Carolina, the Department of Environmental Quality (NCDEQ) issues permits to facilities with at least 250 pigs under the Swine General Permit,^32^ including those not covered by NPDES. Air and water quality issues are coupled, as a major source of pollution from CAFOs with swine is the standard use of open waste cesspits, called lagoons, and spray-based irrigation of this waste onto nearby fields referred to as sprayfields. Industrial practices of manure irrigation use sprinklers to deliver thousands of gallons of fluidized waste per hour to fields and adjacent lands. Although not allowed under the Swine General Permit, residents report that overspray from sprayfields deposits contaminants and waste particles onto their property and persons.^8^ Manure irrigation occurs more frequently in North Carolina than other U.S. states, as cesspits should be maintained near their minimum levels because of the potential for rainstorms.^33,34^ Volatile and semivolatile gases, including those with strong odors such as NH_3_, are emitted to the atmosphere from the cesspits and manure sprayed fields by evaporation. NH_3_ is produced when nitrogen compounds in swine wastes are microbially metabolized or abiotically hydrolyzed. Cesspits have a regularized shape and distinctive pink to brown color, which originates from the high organic matter content rather than any material covering, and have been identified using satellite images and machine-learning techniques.^35,36^

While there are limited routine surface measurements of CAFO-related air quality,^37^ NH_3_ is observed from space by various instruments,^38−41^ including the Infrared Atmospheric Sounding Interferometer (IASI).^42−45^ Satellite-based total NH_3_ vertical column densities combined with oversampling and other superresolution techniques have produced evidence of elevated NH_3_ air pollution over individual CAFOs and quantitative estimates of NH_3_ emission rates, typically after rotating images to a common wind direction.^46−48^ Satellite NH_3_ columns were evaluated using spatiotemporally coincident in situ aircraft profiles over agricultural regions in Central California^49^ and Northeastern Colorado.^50^ Guo et al.^50^ reported that IASI columns and vertically integrated aircraft profiles within the atmospheric boundary layer (ABL) exhibit a linear regression slope of 1.0 ± 0.19 and correlation coefficient of 0.57 using the artificial neural network for IASI (ANNI) retrieval version 3 with meteorological inputs from the European Centre for Medium-Range Weather Forecasts (ECMWF) Re-Analysis (ERA)-Interim. Such comparisons are made difficult by sampling issues that affect in situ NH_3_ techniques, as NH_3_ deposits and is later revolatized from the internal surfaces of instrumentation as a function of gas-phase and surface-adsorbed NH_3_ concentrations and thermodynamic variables.^49,50^ These are the same physical processes of deposition and volatization that affect atmospheric NH_3_ distributions.^51−53^ IASI NH_3_ columns have also been evaluated against ground-based columns using Fourier-transform spectroscopy (FTS)^54−56^ and time-integrated surface measurements from the National Atmospheric Deposition Program Ammonia Monitoring Network,^50,57^ with satellite instruments, including IASI, capturing similar spatiotemporal trends as measured from the surface.

Quantitative analyses of distributive air pollution inequalities associated with CAFOs have relied on indirect approaches of aggregated residential proximities to permitted facilities^1,10,58^ and modeled air pollution concentrations.^9,12^ Satellite NH_3_ measurements reflect spatiotemporal variability in CAFO-related air quality impacts, and here we use IASI NH_3_ columns to report the first observationally based NH_3_ inequalities for Black and African Americans, Hispanics and Latinos, and American Indians across Eastern North Carolina. We investigate variability in NH_3_ distributions with wind speed and air temperature, conditions that affect NH_3_ emissions, mixing, and bidirectional surface exchange and over the full IASI record (2008–2023). We explore variations in distance-dependent relationships between NH_3_ columns and permitted swine facilities, producing empirical constraints on environmental controls over the spatial extent of CAFO-related air pollution exposures. IASI NH_3_ columns are described in policy-relevant contexts, which for pollutants without federal National Ambient Air Quality Standards (NAAQS), consist of state and local regulations and practices and court judgments and settlements. In particular, we focus on outcomes relevant to the IASI observations from the 2018 Settlement Agreement between NCDEQ and the North Carolina Environmental Justice Network, Rural Empowerment Association for Community Help, and Waterkeeper Alliance, Inc., who filed a complaint with the U.S. Environmental Protection Agency (EPA) Office of Civil Rights (now the External Civil Rights Compliance Office) alleging NCDEQ violated Title VI of the Civil Rights Act of 1964^59^ by discriminating against residents in the permitting swine CAFOs on the basis of their “race, color, or national origin”.

## Measurements

### IASI

IASI is an infrared sounder providing NH_3_ observations from onboard various polar-orbiting MetOp satellites at 9:30 am and 9:30 pm local solar time (LT).^42^ NH_3_ is retrieved by fitting absorption features over 812–1126 cm^–1^ and using meteorological inputs from the ECMWF ERA5^44^ and an artificial neural network to transform hyperspectral range indices into total NH_3_ vertical column densities, separately over land and sea scenes.^45,60,61^ Pixels with erroneous spectra and/or heavy cloud coverage are removed prior to the retrieval. We use Level 2 IASI NH_3_ columns based on the current version of the retrieval (IASI NH3R-ERA5 version 4.0.0R, where “R” indicates this is the reanalyzed product as opposed to near real-time retrieval) and available from MetOp-A and B satellites over 1 October 2007–15 October 2021 and 8 March 2013–31 March 2023, respectively, which include improved temporal consistency and a low bias correction of 15–20% over polluted scenes compared to the previous version.^62^ We applied the recommended postfilter to remove columns with clouds and/or limited sensitivity from low thermal contrast.^44^ IASI pixels are circular and 12 km in diameter at nadir and elliptical otherwise.

We average multiple years of morning (9:30 am LT) April–August columns to 0.01° × 0.01° (∼1 km × 1 km) using an oversampling algorithm well-tested for IASI NH_3_ columns in Northeastern Colorado, a location that also has CAFOs.^63^ Following Sun et al.,^63^ IASI pixels are represented using a smooth spatial sensitivity distribution of a two-dimensional standard Gaussian function (exponent of 2) rather than the true super-Gaussian IASI pixel spatial response (exponent of ∼18). IASI pixels are weighted by their uncertainties, including sensitivities to thermal contrast during oversampling. Oversampled NH_3_ columns generally subsample block groups in rural Eastern North Carolina, which are on average 20 km^2^ across the region. Block groups are subdivisions of census tracts containing 600–3000 people or 240–1200 housing units and are the smallest area unit for which the U.S. Census Bureau reports all demographic information. We focus on oversampled morning NH_3_ columns from MetOp-A over April–August 2016–2021. We also separately oversample morning NH_3_ columns on days with mean morning (8 am–12 pm local time, LT) surface wind speeds or air temperatures below (calm or cool conditions, respectively) and above (windy or hot conditions) median morning wind speeds/air temperatures using measurements from the Automated Surface Observing System/Automated Weather Observing System over April–August 2016–2021 (Figure S1). To describe trends in NH_3_ columns over the IASI record, we oversample morning NH_3_ columns from MetOp-A in April–August in 2008–2010, 2011–2013, 2014–2017, and 2018–2021 and from MetOp-B in April–August in 2014–2017, 2018–2021, and 2022–2023 (Table S1).

### Block Group-Scale ΔNH_3_ Inequalities

NH_3_ column enhancements (ΔNH_3_ columns) are calculated as oversampled NH_3_ columns above, and then less, the tenth percentile of the column distribution across North Carolina for a given period (Table S1). The tenth percentile was determined empirically and selected because it was found to be the highest decile yielding statistically equivalent absolute inequalities in NH_3_ and ΔNH_3_ columns (Table S2). This constraint is based on the physically realistic assumption that block group-scale variability is not driven by variations in the tropospheric NH_3_ background on average. We focus on block groups with ΔNH_3_ columns. Differences in inequalities in NH_3_ and ΔNH_3_ columns at higher deciles are a sampling effect caused by changes in the underlying population distribution. Area-weighted mean ΔNH_3_ columns were computed within block group polygons. The process workflow is presented in SI*Appendix 1*. For the different time periods of study, we computed corresponding tropospheric NH_3_ tenth-percentile backgrounds using oversampled columns from each period. For the ΔNH_3_ columns sorted by wind speed and air temperature in April–August 2016–2021, we used the same tropospheric NH_3_ background as for the ΔNH_3_ columns on all days in that period (Table S1). As an example, the tropospheric NH_3_ background in April–August 2016–2021 is 3.8 × 10^15^ molecules cm^–2^, with ΔNH_3_ columns in almost all (99%) Eastern North Carolina block groups.

We report NH_3_ inequalities as equal to the absolute and relative (percent) differences between population-weighted ΔNH_3_ columns (eq S1) for non-Hispanic/Latino Black and African Americans, referred to as Black and African Americans, Hispanics and Latinos of all races, referred to as Hispanics and Latinos, and non-Hispanic/Latino American Indians/Alaska Natives, referred to as American Indians, compared to non-Hispanic/Latino whites. Inequalities are based on the subset of block groups with populations for a given group equal to or greater than the mean across all Eastern North Carolina block groups (Figure S2). Mean block group-scale populations in Eastern North Carolina counties are Black and African Americans, 25%; Hispanics and Latinos, 10%; American Indians, 2%; and non-Hispanic/Latino whites, 56%. Uncertainties in block group-scale inequalities are reported as standard mean errors. Race and ethnicity data are from the U.S. Census 2020 decennial census. Because there are concerns that multiple marginalized population groups were significantly undercounted in the 2020 decennial census, which is currently unresolved in the dataset, we compared results between the 2020 decennial census and 5-year 2016–2020 American Community Survey (ACS). We calculate slightly higher relative and absolute NH_3_ inequalities using the 2020 decennial census; however, differences are not statistically significant (Table S3). Inequalities in 2008–2010 and 2011–2013 are computed using both the 2010 and 2020 decennial census as described below.

### Surface Winds and Air Temperatures

Hourly meteorological measurements are available from the Automated Surface Observing System and Automated Weather Observing System and accessible through the Iowa State University Iowa Environmental Mesonet download service.^64^ Corresponding to the 9:30 am IASI overpass, we calculate mean morning (8 am–12 pm LT) surface wind speeds and air temperatures and mean daily (24 h) total precipitation from 38 monitors in Eastern North Carolina counties (Figure S3). Not all monitors have data in all years.

### NCDEQ Permitted Animal Facilities

The NCDEQ permits Animal Feeding Operations (AFOs), defined as facilities with more than 250 swine, 100 confined cattle, 75 horses, 10^3^ sheep, or 3 × 10^4^ poultry with liquid waste management.^65^ AFO location, allowable animal count and type, and number of waste cesspits (for swine) are made publicly available. Most swine facilities are also permitted under the 5-year North Carolina Swine Waste Management System General Permit, referred to as the Swine General Permit.^32^ We use the most recent NCDEQ AFO database, dated 4 May 2023.^66^

## Results and Discussion

### NH_3_ Inequalities

**Figure 1:**
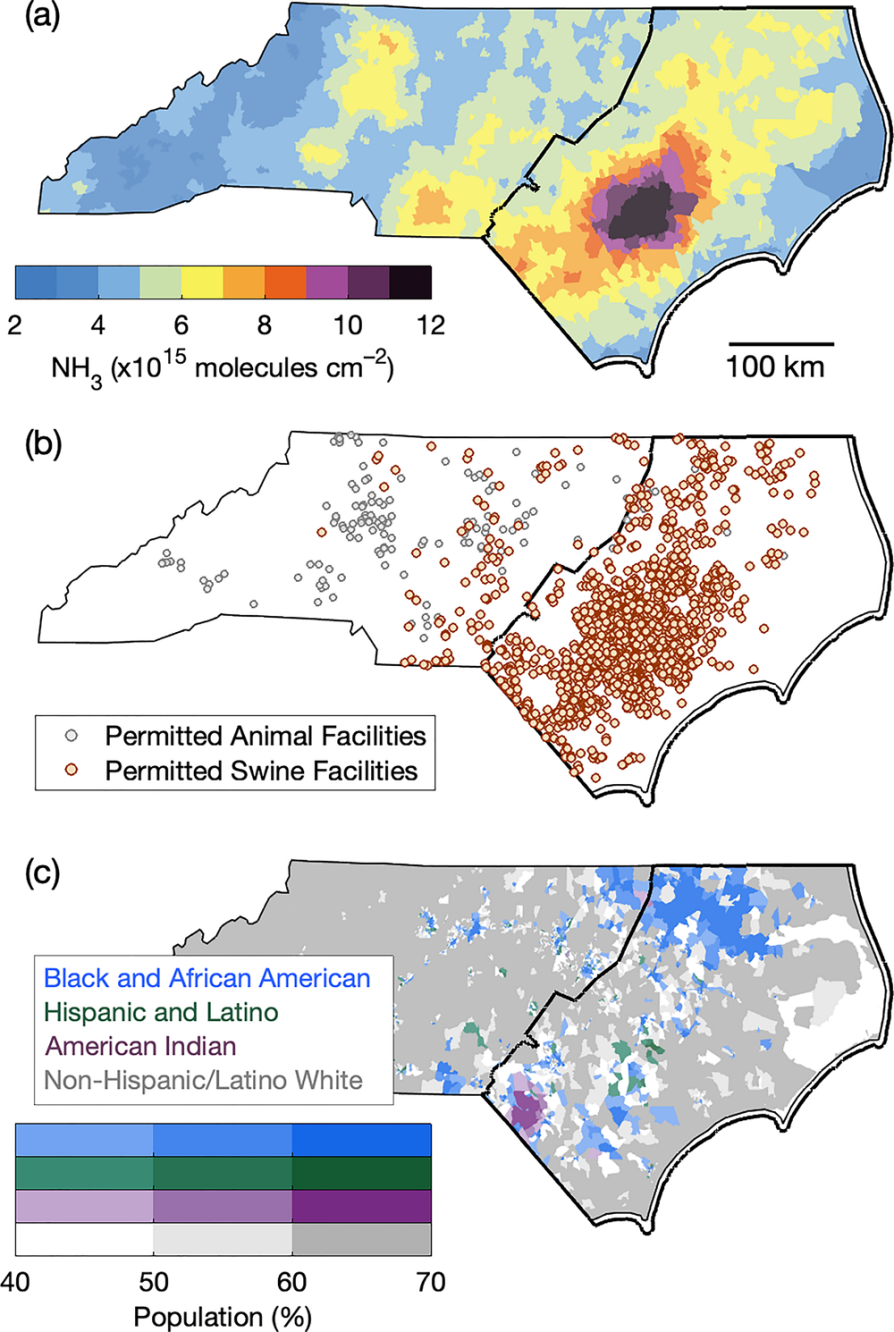
Block group-scale NH_3_ columns (molecules cm^–2^) in April–August 2016–2021 (a), 2023 NCDEQ permitted animal (gray) and swine (brown) facilities (b), and population of the majority race-ethnicity group in each block group (%): Black and African American (blue), Hispanic and Latino (green), American Indian (purple), and non-Hispanic/Latino white (gray) (c).

NH_3_ columns are elevated in Eastern North Carolina, especially where there are numerous permitted swine facilities (Figure 1), including in Sampson and Duplin Counties, parts of Wayne, Lenoir, Bladen, Greene, and Jones Counties, and counties home to the Coharie, Lumbee, and Waccamaw Siouan Indian Tribes. We report block group-scale NH_3_ differences over 2016–2021 (Table 1), focusing on April–August because this is when NH_3_ columns are highest (Figure S4), as NH_3_ emissions are temperature dependent,^67−69^ and surface-air thermal contrasts in the lower troposphere are maximized, producing NH_3_ column observations that are more accurate.^44^ Because the lifetime of gas-phase NH_3_ is hours to days,^70^ the highest NH_3_ concentrations are colocated with emission sources in the ABL, and, potentially, near-surface nocturnal residual layers that are reincorporated into the ABL the following morning.^71−73^ NH_3_ can be present at pptv-to-low ppbv levels in the free troposphere and background ABL, where it is more evenly distributed spatiotemporally.^45,74−76^ This has the effect of reducing observed relative, but not absolute, differences in surface-level NH_3_ in total NH_3_ columns, motivating our use of ΔNH_3_ columns.

**Table 1:** Mean Block Group-Scale Relative and Absolute Inequalities in ΔNH_3_ Columns in Eastern North Carolina for Black and African Americans, Hispanics and Latinos, and American Indians Compared to Non-Hispanic/Latino Whites: All Days (April–August 2016–2021), All Days (May–July 2016–2021), Days with Morning (8–12 am LT) Surface Wind Speeds Below (Calm) and Above (Windy) the Median in April–August 2016–2021, and Days with Morning Surface Air Temperatures Below (Cool) and Above (Hot) the Median over the Same Period^a^

	relative ΔNH_3_ inequality (%)			absolute ΔNH_3_ inequality (×10^14^ molecules cm^–2^)		
	Black and African Americans	Hispanics and Latinos	American Indians	Black and African Americans	Hispanics and Latinos	American Indians
April–August 2016–2021	27 ± 3	35 ± 3	49 ± 3	6.3 ± 0.6	8.4 ± 0.8	12.7 ± 0.8
May–July	21 ± 2	25 ± 3	31 ± 3	6.5 ± 0.7	8.2 ± 1.0	10.5 ± 0.9
calm days	27 ± 3	36 ± 4	64 ± 4	7.3 ± 0.8	10.0 ± 1.0	21.3 ± 1.1
windy days	21 ± 3	28 ± 3	24 ± 3	4.4 ± 0.6	6.0 ± 0.7	5.2 ± 0.7
cool days	18 ± 3	28 ± 3	55 ± 3	3.6 ± 0.5	5.9 ± 0.7	13.9 ± 0.8
hot days	31 ± 3	36 ± 4	31 ± 4	8.7 ± 0.9	10.7 ± 1.2	8.9 ± 1.1
April–August (physics-based oversampling)	22 ± 3	31 ± 3	45 ± 3	5.4 ± 0.7	7.7 ± 0.9	12.5 ± 0.9

a Also shown: mean block group-scale relative and absolute inequalities in ΔNH_3_ columns April–August 2016–2021, oversampled using a physically-realistic IASI spatial response function. Uncertainties are standard mean errors.

Block group-scale ΔNH_3_ columns are 27 ± 3% higher for Black and African Americans, 35 ± 3% higher for Hispanics and Latinos, and 49 ± 3% higher for American Indians compared to non-Hispanic/Latino whites in April–August 2016–2021, demonstrating systematic inequalities in atmospheric NH_3_ concentrations in Eastern North Carolina (Table 1). This comparison is based on population-weighted ΔNH_3_ columns in block groups where the population of that group is equal to or above the mean population across Eastern North Carolina counties. Population-weighted NH_3_ column inequalities across all block groups are also statistically significant: Black and African Americans, 15 ± 2% (3.7 ± 0.4 × 10^14^ molecules cm^–2^); Hispanics and Latinos, 19 ± 2% (4.6 ± 0.5 × 10^14^ molecules cm^–2^); and American Indians, 34 ± 2% (8.8 ± 0.4 × 10^14^ molecules cm^–2^). Uncertainties are based on standard mean errors, with precision improving with spatial averaging using population weighting over many block groups.

NH_3_ is a documented atmospheric emission from CAFOs, and elevated NH_3_ mixing ratios have been measured and modeled in the vicinity of regional swine facilities.^16,77,78^ Researchers and the NCDEQ have already shown there are more permitted animal facilities near the residences and schools of Black and African Americans, Hispanics and Latinos, and American Indians in Eastern North Carolina counties and statewide.^1,9,10,58,79^ That said, CAFOs are not the only source of atmospheric NH_3_. NH_3_ also emitted following application of anhydrous ammonia or ammonium salts or biogeochemical processes in fertilized fields. Fertilizer includes both fixed nitrogen and animal wastes. Where used, fertilizer application is recommended in the spring (mid-February–March) and/or fall (mid-August–September).^80^ To minimize the contribution of crop agriculture on ΔNH_3_ columns, we also compute NH_3_ inequalities in May–July (Table 1). Absolute NH_3_ inequalities in May–July are statistically equivalent to those in April–August, but relative inequalities are 6–18 ± 4 points lower. While ΔNH_3_ columns are higher regionally in May–July, NH_3_ spatial heterogeneities are similar, suggesting the same emissions sources drive the inequalities in both periods. Because the NH_3_ distribution and abundance are also affected by surface winds, temperature, and precipitation, we test whether these conditions differ on average between May–July and April–August in 2016–2021. Mean surface wind speeds are equal in May–July and April–August (3 ± 0.1 m s^–1^, errors as standard mean errors) and air temperatures are slightly higher in May–July (24.9 ± 0.2 °C) than in April–August (23.7 ± 0.2 °C) in 2016–2021. NH_3_ is water-soluble and removed from the atmosphere by wet deposition, but mean daily precipitation totals are 0.14 ± 0.01 mm in both May–July and April–August 2016–2021.

Wind and air temperature are physical controls on NH_3_ concentrations and, therefore, NH_3_ inequalities and exposures. Dispersion is a major factor influencing the distribution of primary pollutants, e.g., NH_3_ and coemitted species, near sources. Dispersion gradients are exponential, with dilution by background air being less efficient when winds are slow. On days with calm (below median) morning surface wind speeds, absolute NH_3_ inequalities for Black and African Americans and Hispanics and Latinos are ∼50% higher than when winds are fast (above the median). For American Indians, absolute NH_3_ inequalities are more than twice as large on calm versus windy days (Table 1). Higher NH_3_ inequalities on calm days are observational evidence of air quality consequences for Black and African Americans, Hispanics and Latinos, and American Indians because they live closer to swine CAFOs on average. Population-weighted distances between block group center points and the nearest permitted swine CAFO in Eastern North Carolina are 8.3 km for Black and African Americans, 7.7 km for Hispanics and Latinos, and 5.7 km for American Indians compared to 10.8 km for non-Hispanic/Latino whites in block groups equal to and above the mean population across Eastern North Carolina.

NH_3_ emissions are also temperature dependent,^67,68^ with temperature affecting the distribution of NH_3_ through multiple processes that broaden the spatial extent of CAFO-related air quality impacts. Briefly, NH_3_ is lost from the atmosphere through deposition and can be subsequently revolatized to the atmosphere as a function of temperature. This process is commonly referred to as the NH_3_ bidirectional flux, and it increases atmospheric NH_3_ concentrations downfield of sources.^51,53,81−84^ The NH_3_ bidirectional flux varies with the relevant concentration difference between the atmosphere and surface, with surface saturation from deposited NH_3_ causing surfaces to potentially be only emissive. Second, when waste management includes applying swine wastes to fields through manure irrigation, a practice of most swine CAFOs,^34^ nitrogen in sprayed fields can become a temperature-dependent NH_3_ source, where sprayfields are typically located within ∼1 km of the cesspits.^33^ Third, NH_3_ is also in thermal equilibrium with particle-phase ammonium, with higher temperatures and lower humidity driving the release of NH_3_. Particles transported downfield during cooler and more humid nights, which also contribute to the air quality impacts of CAFOs, release gas-phase NH_3_ when temperatures warm in the morning.^85−87^ Absolute NH_3_ inequalities for Black and African Americans and Hispanics and Latinos are 83% and 58% larger, respectively, on hot than cool days. By contrast, absolute NH_3_ inequalities for American Indians are 50% higher on cool days. Because American Indians live closer on average to NH_3_ sources, the largest inequalities are under conditions that localize NH_3_ in relation to the source. Higher absolute NH_3_ inequalities on hot days for Black and African Americans and Hispanics and Latinos imply these groups are additionally affected by processes and practices in which temperature-dependent NH_3_ emissions and concentrations are spatially distributed away from swine CAFOs.

**Figure 2:**
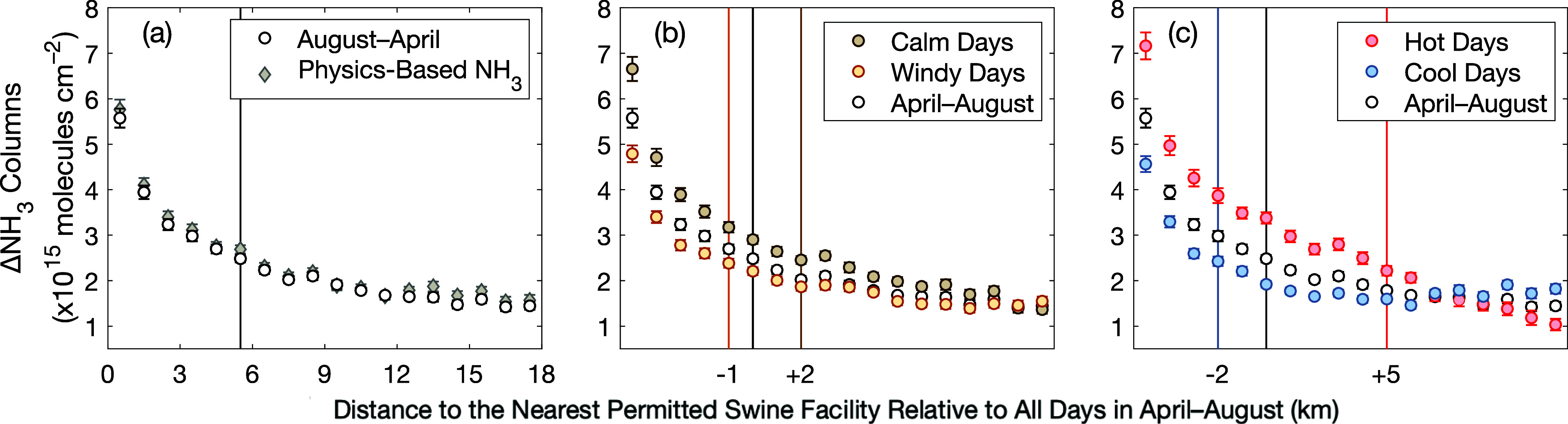
Block group-scale ΔNH_3_ columns as a function of their distance to the nearest NCDEQ permitted swine facility relative to all days over April–August 2016–2021, both with columns oversampled assuming a smooth two-dimensional standard Gaussian function (black circles) and the IASI super-Gaussian sensitivity distribution as the spatial response (gray diamonds) (a), separately on calm (brown) and windy (orange) days (b), and on cool (blue) and hot (red) days (c). Data from panel (a) are shown in panels (b, c) for reference. Error bars are standard mean errors. Vertical lines have a signal-to-noise ratio of 2, with noise defined by the 2σ standard deviation at distances of 15–20 km over all days with observations in April–August 2016–2021.

ΔNH_3_ columns correspond spatially with the locations of permitted swine facilities, based on block group center points within 1 km of a facility address, with ΔNH_3_ columns decreasing exponentially with increasing distance from the nearest swine CAFO (Figure 2). Spatial gradients are a combined function of real variability in the NH_3_ distribution and the convolution of IASI pixels that are larger than the oversampling grid and their spatial response function.^63^ This has the effect of biasing oversampled columns directly over the source, and their derived inequalities,^88^ low, with a portion of the observed downfield impacts being an artifact of the averaging. The spatial resolution of oversampled NH_3_ columns affects the sampling density but is limited by the IASI pixel size. Still, oversampling produces NH_3_ columns that better distinguish concentration hotspots than other techniques.^63^ ΔNH_3_ columns oversampled with a smooth two-dimensional standard Gaussian function and a physics-based approach using the IASI super-Gaussian sensitivity distribution as the spatial response yield similar relative and absolute inequalities and downfield decay-gradients (Table 1 and Figure 2). While there is a tendency for lower inequalities with the physics-based IASI pixel spatial response, as NH_3_ inequalities are driven in large part by proximity to NH_3_ sources, these inequalities are equal to within associated uncertainties. Use of the smooth two-dimensional standard Gaussian function is common practice for IASI^89^ and other satellites observations^90^ that are noisy and sparse that can yield noisy and unphysical results and obscure localized concentration hotspots.^63^

The NH_3_ spatial distribution influences residents’ exposures to NH_3_ and other CAFO emissions. Because a portion of the observed gradient is derived from the pixel spatial convolution, we are cautious not to make claims about the exact value of the NH_3_ length scale with respect to the nearest permitted swine facility on all days over April–August 2016–2021 (which we compute to be 5 km). However, pixel spatial convolution is independent of environmental conditions that affect real variability in the NH_3_ spatial distribution. We quantify the downfield spatial extent based on a signal-to-noise ratio of 2, with the signal as mean ΔNH_3_ columns with uncertainties as standard mean errors and noise defined by the 2σ standard deviation in ΔNH_3_ columns at distances of 15–20 km over all days with observations in April–August 2016–2021 (Figure S5). This is a conservative estimate, with higher mean ΔNH_3_ columns apparent even further away. On days with calm (below median) morning surface wind speeds, we observe higher ΔNH_3_ columns over permitted swine facilities and ΔNH_3_ columns that remain elevated over the regional background for 2 km further downwind than on all days (April–August). On windy days, ΔNH_3_ columns are comparatively lower and decay to the regional background on shorter length scales. We observe the highest ΔNH_3_ columns over permitted swine facilities on hot days, defined as days above median morning temperatures in April–August. ΔNH_3_ columns remain elevated 5 km further downfield of permitted swine facilities than on all days, varying more linearly than exponentially (Figures 2 and S5). This reflects a source term in addition to direct emissions and dispersion, consistent with NH_3_ volatization downfield of swine CAFOs. Observed gradient variability demonstrates residents living multiple kilometers from swine facilities are chronically, i.e., on average on calm and/or hot days, constituting more than half of all days in April–August, exposed to NH_3_ concentrations elevated over the regional mean.

Pinder et al.^77^ reported consonant average spatial correlations between satellite NH_3_ columns from the Tropospheric Emission Spectrometer (TES) and total CAFO number within 10 km of a satellite pixel in Eastern North Carolina, observing even steeper NH_3_ gradients in surface measurements from two-week integrated samplers along the TES flightpath. TES has a higher spectral and spatial (5 km × 8 km) resolution, although lower spatial coverage than IASI.^39^ Likewise, Wilson and Serre^16^ measured NH_3_ mixing ratios using two-week integrated passive diffusion tube samplers in Duplin and Greene counties to be almost twice as high within 0.5 km than 0.5–1 km of a swine facility. Because of their pixel size and the application of oversampling, multiyear mean IASI ΔNH_3_ columns underestimate NH_3_ over and in the very near-field of facilities, leading to IASI-based inequality estimates that are biased low. Techniques combining superresolution algorithms with plume rotation have been shown to enhance the spatial detail in IASI columns and used to quantify NH_3_ emissions from individual CAFOs.^46−48^ This is an aspatial approach not suitable for describing NH_3_ distributive inequalities. In Eastern North Carolina, such techniques are further challenged by the density of CAFOs, as NH_3_ plumes from adjacent facilities overlap. High-time resolution columns measured by FTS found multifold higher NH_3_ near dairy and cattle CAFOs in Northeastern Colorado, with 50% of NH_3_ variability observed within 2 km and 90% within 6 km of facilities.^56^ These length scales reinforce conclusions of a low bias in IASI-based inequalities. Finally, morning IASI sampling times likely further bias NH_3_ columns and absolute inequalities low. NH_3_ columns from the satellite-based Cross-track Infrared Sounder (CrIS), which has an early afternoon overpass and improved surface sensitivity over IASI, are consistently higher than IASI NH_3_ columns.^91,92^ However, CrIS NH_3_ columns are not ready for wide public use. At the same time, IASI provides smaller pixels, a longer time record, and sensors on more satellites.

### NH_3_ and ΔNH_3_ Columns over Time

**Figure 3:**
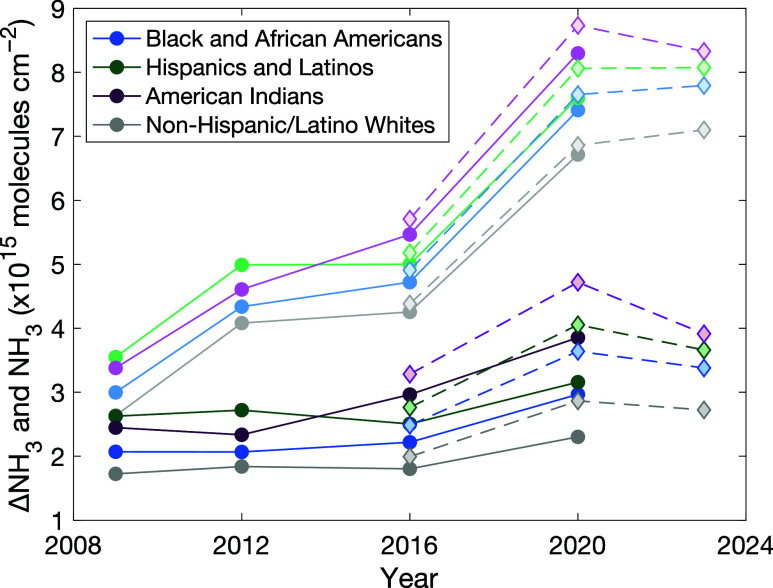
Population-weighted, block group-scale NH_3_ (pastel) and ΔNH_3_ columns (bright) in Eastern North Carolina for Black and African Americans (blue), Hispanics and Latinos (green), American Indians (purple), and non-Hispanic/Latino whites (gray). IASI observations from MetOp-A (circles, solid line) in April–August in 2008–2010, 2011–2013, 2014–2017, and 2018–2021 and MetOp-B (diamonds, dashed line) in April–August in 2014–2017, 2018–2021, and 2022–2023. Standard mean errors are similarly sized as the markers and omitted for clarity.

IASI NH_3_ columns inform multiyear trends in NH_3_ spatial distributions over 2008–2023. NH_3_ columns collected by IASI instruments are available with the most recent retrieval from the MetOp-A and B satellites. To interpret NH_3_ columns over the full IASI record, we first compare IASI MetOp-A and B observations in their coinciding windows of 2014–2017 and 2018–2021 (Table S4). Even though population-weighted ΔNH_3_ and NH_3_ columns from MetOp-B are slightly systematically higher than those from MetOp-A, trends in MetOp-A and B-based columns are consistent (Figure 3). Additionally, population-weighted IASI ΔNH_3_ columns from both satellites produce relative NH_3_ inequalities equal to within associated uncertainties. Absolute NH_3_ inequalities for MetOp-A and B are also statistically equivalent for all groups in 2014–2017 and for Black and African Americans in 2018–2021. Absolute NH_3_ inequalities from MetOp-B are similar but slightly larger than observed from MetOp-A for Hispanics and Latinos and American Indians in 2018–2021.

Population-weighted NH_3_ columns increased substantially (∼50%) over 2008–2023; however, smaller changes are observed in ΔNH_3_ columns (Figure 3). This implies that multiyear trends in NH_3_ columns in Eastern North Carolina are largely driven by controls affecting the regional distribution of NH_3_ rather than localized changes in NH_3_ emissions. Briefly, in the 1980s and 1990s, industrial swine facilities concentrated in Eastern North Carolina through processes of farm consolidation, industrial land grabbing, and state legislation providing incentives and preventing localities from addressing offensive odors with zoning.^93,94^ A temporary moratorium was placed on new swine CAFOs in 1997, with a ban on new lagoons and mandates around infrastructure in new and/or expanding CAFOs becoming permanent in 2007.^95^ While these events predate the IASI NH_3_ record, observed trends in the number of waste cesspits detected from space^36^ and measurements of rainfall dissolved ammonium,^96^ which can be scavenged as NH_3_ or particle-phase ammonium, are generally consistent with this timeline. ΔNH_3_ columns have been similar over the IASI record, indicating persistent, unresolved, and unequal CAFO-related NH_3_ air quality impacts since 2008. Relative and absolute NH_3_ inequalities for Black and African Americans and Hispanics and Latinos have been steady within associated uncertainties over 2008–2023. We observe more variability in inequalities for American Indians, with statistically significant increases and decreases in relative and absolute NH_3_ inequalities in 2014–2017 and 2022–2023. Multiyear trends in ΔNH_3_ columns have a much larger effect on inequalities than changes in population composition (Figure S6). Lastly, trends in NH_3_ columns are caused by a shift in the full distribution of observations, with increases in both mean NH_3_ columns across population groups and empirically determined tropospheric NH_3_ background column densities. Multiyear trends in NH_3_ columns have therefore been influenced by climatological and/or secondary processes^97^ that do not substantially affect ΔNH_3_ columns and block group-scale inequalities. We test for corresponding trends in mean morning surface air temperatures but find they varied by less than 0.3 °C in April–August over 2008–2023. In addition, IASI columns use a consistent retrieval process across all years. This then supports explanations in the literature for other locations^97−101^ that increases in NH_3_ columns in Eastern North Carolina are driven by ongoing emissions reductions in sulfur dioxide and nitrogen oxides as opposed to localized changes in NH_3_ emissions, affecting NH_3_ concentrations more evenly spatially. This is because of the time required for chemistry, that the lifetime of ammonium sulfate and nitrate against deposition is much longer (∼7–10 days) than NH_3_ (∼1 day), and the potential for ammonium nitrate to rerelease gas-phase NH_3_. That said, increases in NH_3_ lifetime from surface saturation by deposited NH_3_ could also partly explain both increased NH_3_ columns and reduced NH_3_ spatial heterogeneities.

### Applications and Policy Relevance

NH_3_ and other CAFO coemissions do not have corresponding EPA NAAQS. This leaves air quality control to a complex web of state and local regulations and legal cases and settlements, if it is done at all. Even though our focus is on North Carolina, some version of this localized decision-making is influential across the U.S. We discuss the IASI NH_3_ columns in this context, which is often overlooked in research on air pollution by scientists. The NCDEQ is the state governmental agency responsible for environmental protection, including swine CAFO permitting. NCDEQ’s recent activities around the unequal air quality impacts of swine CAFOs have largely been required by a negotiated settlement with community organizations seeking environmental justice for environmental racism in Eastern North Carolina. Here, we describe the outcomes of this settlement, as well as related regulatory guidance, activities, and judgements, focusing our discussion on the aspects to which IASI NH_3_ columns are relevant.

In 2014, the North Carolina Environmental Justice Network (NCEJN), Rural Empowerment Association for Community Help (REACH), and Waterkeeper Alliance, Inc. submitted a complaint to the now U.S. EPA External Civil Rights Compliance Office (ECRCO) alleging that industrial swine permitting and pollution disproportionately affected Black, Latino, and American Indian residents, violating Title VI of the 1964 Civil Rights Act.^102^ In 2016, these organizations filed a second complaint claiming NCDEQ engaged in and failed to protect residents involved in the 2014 complaint from intimidation and threats of violence.^103^ In 2017, the EPA sent a Letter of Concern to the NCDEQ, providing preliminary information on ECRCO’s investigation describing evidence supporting residents’ claims.^8^ The NCDEQ, NCEJN, REACH, and Waterkeeper Alliance, Inc. entered into mediation, reaching their Settlement Agreement in 2018. As part of this Settlement Agreement, the NCDEQ was obligated to complete the so-named Duplin County Air Monitoring Study, revise the Swine General Permit with community input, change its Title VI compliance programs, and develop an environmental justice mapping tool.^104^ IASI NH_3_ columns provide insight around these activities and their impacts, and we discuss them here in turn.

Briefly, the NCDEQ Division of Air Quality (DAQ) conducted the Duplin County Air Monitoring Study largely by measuring NH_3_, H_2_S, and fine particulate matter (PM_2.5_) at two locations in Duplin County over October 2018–October 2019.^105^ DAQ focused on PM_2.5_ NAAQS exceedances; however, because NH_3_ and H_2_S do not have NAAQS, compliance standards were based on the North Carolina Acceptable Ambient Levels (NC AALs).^106^ The NH_3_ NC AAL for a 1 h Acute Irritant is 2.7 mg m^–3^ or 3.868 ppm, which is the only NC AAL for NH_3_.^105^ NC AALs are concentration-based emissions limits on industrial stationary sources such that ambient levels cannot exceed the AAL. NC AALs apply to individual facilities and are not comparable to measured atmospheric mixing ratios.^106^ DAQ located NH_3_ instrumentation at a minimum of 0.8 km from the nearest permitted swine facility following EPA community-oriented monitoring requirements.^107^ EPA siting requirements are intended to reduce the influence of any one facility, producing measurements representing air quality for communities broadly. Because NC AALs must be met at the property boundary, dispersion models are used for observations collected beyond the fenceline;^106^ although, this modeling was never published. During the Duplin County Air Monitoring Study, DAQ reported ambient NH_3_ mixing ratios of 0 ppb during almost all hours of the year and no exceedances of the NC AALs.^105,108^ Consequentially, NCDEQ concluded there were no significant air quality issues and no further monitoring was recommended.

How then do we reconcile existing research on swine CAFO emissions, their attendant health impacts, and the embodied sensing of residents, which includes nausea and vomiting, illness and disease, stress, and feelings of being a prisoner in one’s own home, with the year-long Duplin County Air Monitoring Study that reported zero NH_3_ pollution at almost all hours? Criticisms of the DAQ study have focused on monitor siting, specifically concerns that NH_3_ instrumentation was located too far away from any given facility to record elevated NH_3_ concentrations.^109^ While EPA community-oriented monitoring requirements are designed to describe air quality on average, swine facilities are ubiquitous in many Eastern North Carolina counties such that measurements at CAFO boundaries are in fact representative of exposures for much of the population. DAQ acknowledged the challenges of meeting EPA guidelines because of the density of swine facilities.^109^ Therefore, DAQ siting decisions were not responsive to the distinct local emissions source distribution characteristics, application of NC AALs, or residents’ expressed priorities and needs. That said, DAQ monitors were located within, although near the edge, of the largest region of enhanced NH_3_ in Eastern North Carolina according to IASI NH_3_ columns (Figures 1 and S7), with comparable daily surface median wind directions and mean wind speeds in April–August in 2019 and 2016–2021 of south–southwest winds at 3.0 ± 0.1 m s^–1^. Elevated ΔNH_3_ columns are also observed farther than 1 km from swine CAFOs, at least average on days with calm winds and/or hot air temperatures (Figure 2). Based on these patterns in the IASI measurements, we conclude that DAQ should have detected at least some level of nonzero atmospheric NH_3_, if not fenceline concentrations, in April–August on average—why did they not?

DAQ quantified NH_3_ using an in situ technique based on electrochemical cell detection developed for industrial monitoring (AreaRAE). The instrument has a detection limit and resolution of 0.1 ppm,^105^ meaning NCDEQ reported 0 ppb NH_3_ while the NH_3_ mixing ratio was some value <100 ppb. Based on previously collected NH_3_ measurements in the region and elsewhere, one would expect NH_3_ mixing ratios over cesspits at low ppm levels^110^ and downfield of facilities commonly at tens of ppb.^16,77^ Combined with the application EPA community-oriented monitoring siting requirements, the technique selected by DAQ was not adequate to resolve known NH_3_ variability. DAQ described multiple nighttime NH_3_ events, including one on 14–16 February at Williamsdale Farm that exceeded 2 ppm, offering a potential glimpse into NH_3_ levels at CAFO boundaries. However, the study’s focus on the NC AAL, applied without the required dispersion modeling, means these events were not considered exceedances. Standards other than the NC AAL were available to the NCDEQ (Table S5). The U.S. Department of Health and Human Services Agency for Toxic Substances and Disease Registry (ATSDR) identifies the acute inhalation standard for NH_3_ respiratory effects to be 1.7 ppm,^111^ which was exceeded during the February event. The ATSDR NH_3_ chronic inhalation standard for respiratory effects is 0.1 ppm,^111^ equal to the AreaRAE detection limit.^105^ Additionally, odor is an air quality and quality of life concern of residents,^8^ with NH_3_ described as pungent and smelling of rotten fish and cat urine. Odor thresholds are variable in part because of the wide range of experimental conditions used to quantify thresholds empirically.^112−114^ The American Industrial Hygiene Association catalogs published odor thresholds and includes published evidence of NH_3_ odor thresholds as low as 43 ppb,^115^ far below the 5 ppm threshold applied by DAQ without caveat.^105^ DAQ measured H_2_S, an NH_3_ coemission, described as smelling like rotten eggs, above odor thresholds frequently during spring and summer months, consistent with the NH_3_ seasonality observed by IASI. CAFOs emit various other gases as well, and NH_3_ observations lower than NH_3_ health or odor thresholds do not prove that CAFO-related air pollution is not a nuisance and/or harmful to residents. However, in the context of IASI NH_3_ columns, measurements of 0 ppb NH_3_ at almost times may indicate the DAQ instruments were not functioning adequately. DAQ has not made public analytical evidence on AreaRAE field performance, e.g., linearity in sensitivity over 0–50 ppm of NH_3_ (the instrument was calibrated with a 50 ppm of NH_3_ standard) or demonstration of the absence of sampling inlet interferences and/or the kinds of cross-sensitivities anticipated for electrochemical sensors.

Second, NCDEQ revised the Swine General Permit with more restrictions on the locations of manure irrigation fields and facility expansions and providing more communication between NCDEQ and concerned residents.^116^ In place since 2019, these modifications do not correspond to decreases in IASI ΔNH_3_ columns (Figure 3), which would reveal an effective process responsive to voiced community preferences for pollution mitigation and elimination of cesspit/sprayfield waste management practices. Here, the data collected by DAQ in the Duplin County Air Quality Study have material consequences, as they evidence the absence of air pollution impacts from swine facilities. The revised Swine General Permit includes minimal changes to enforcement methods and no new management or infrastructure requirements around air emissions. NCDEQ adopted new procedures for receiving and investigating residents’ complaints. However, while NCDEQ received hundreds of complaints annually about CAFO-related odors in the 1990s, fewer than 30 complaints were received in the last five years.^116^ NCDEQ claimed this decline was due to pollution control through the Swine General Permit,^116^ but multiyear trends in IASI ΔNH_3_ columns indicate no reduction in CAFO-related air quality impacts since 2008.

Finally, the NCDEQ adopted EPA guidelines to designate Potentially Underserved block groups across North Carolina^117^ and created a community mapping tool to inform some decision-making e.g., outreach plans.^118^ Potentially Underserved block groups (Figure S8) are defined as the approximately 25% of North Carolina block groups where (a) at least 50% of residents did not identify in the U.S. Census as non-Hispanic/Latino white or the population of Black and African American, Hispanic and Latino, American Indian, Asian, and mixed-race residents is >10% higher than the county and/or state mean and (b) at least 20% of residents are below the federal poverty line or the portion of households below the poverty line is >5% higher than the county and/or state mean.^119^ ΔNH_3_ columns are 20 ± 3% higher in Potentially Underserved than other Eastern North Carolina block groups. Compared to NH_3_ inequalities based on population-weighted ΔNH_3_ columns as defined here (Table 1), decision-making based on Potentially Underserved block groups will inadequately respond to environmental racism in CAFO-related air quality impacts, as NH_3_ inequalities are, by comparison, higher for Black and African Americans, Hispanics and Latinos, and American Indians in Eastern North Carolina on average. The NCDEQ Community Mapping System visualizes the spatial correspondence between Potentially Underserved block groups, Tribal Community boundaries, NCDEQ AFO and NPDES permits, and other point sources (although only the Potentially Underserved block groups and Tribal Community boundaries were viewable at the time of writing).^118^ As air quality impacts are contested, IASI NH_3_ columns, even without resolving individual facilities, provide observational evidence of systematic NH_3_ inequalities relevant to the issue that are left open to dispute in the current mapping tool.

### Implications and Considerations

IASI ΔNH_3_ columns identify distributive NH_3_ inequalities across Eastern North Carolina, with space-based measurements collected routinely in the absence of surface monitoring. IASI ΔNH_3_ columns provide observational constraints on the environmental variability and spatial extent of CAFO-related air pollution impacts: residents living multiple kilometers from the nearest swine CAFO are exposed to elevated NH_3_ in April–August; relationships with wind speed imply exponentially higher NH_3_ at facility boundaries and more disproportionate impacts when winds are calm; and NH_3_ distributions are temperature-dependent, with NH_3_ volatization away from facilities, e.g., from manure-sprayed fields and particles, worsening inequalities for Black and African Americans and Hispanics and Latinos. IASI is thus well positioned to monitor CAFO-related NH_3_ inequalities, including in areas without the CAFO location information that is unavailable in most states. We note that inequalities in ΔNH_3_ columns are likely a lower bound, as dispersion-decay gradients are steeper than IASI pixels even with oversampling,^12,16,77^ IASI measurements are collected in the morning (and night), and considerable time averaging is required to reduce associated noise. Finally, the IASI NH_3_ columns are unlikely to resolve whether some management practices cause lower atmospheric NH_3_ than others within Eastern North Carolina. This is a combined function of IASI’s analytical limitations, the dense clustering of swine facilities in the region, and the limited public records with which to subset permitted swine facilities; therefore, we do not draw related conclusions. What does emerge in this analysis, is evidence that decision-making relevant to swine CAFOs and NH_3_ inequalities has failed to materially address residents’ claims and experiences of harm. For environmental justice defined as remedy for environmental racism, discussion and advocacy of specific agricultural practices and their benefits should foreground the preferences of affected residents.

NH_3_ concentrations are spatially and temporally heterogeneous, horizontally and vertically, making it challenging to derive surface mixing ratios from NH_3_ columns for the application of health and odor thresholds. An analysis based on NH_3_ columns as opposed to surface mixing ratios is justified for three reasons: variability affecting aggregate block group-scale NH_3_ inequalities is driven by surface-level processes; there are no NH_3_ NAAQS or other health-based concentration standards the exceedance of which trigger specific regulatory intervention, enforcement, or particularized benefits for affected residents; and NH_3_ columns are consistent with testimonies from Eastern North Carolina residents that CAFO-related air quality impacts corresponding to these column densities causes them harm.^8^ Estimates of NH_3_ surface levels are highly uncertain because of the lack of temporally coincidental measurements in the region, which, relatedly, has been used to deny residents’ claims around atmospheric exposure and odor. Relevant to analyses of distributive inequalities to inform decision-making is whether spatial patterns in NH_3_ columns reflect those at the surface, not the inequalities in the mixing ratios themselves. This correspondence is expected based on the length scales of NH_3_ gradients and past research on satellite nitrogen dioxide inequalities.^88,120,121^ Finally, estimates of NH_3_ mixing ratios may not advance knowledge around CAFO-related air quality impacts or residents demands for environmental justice more than NH_3_ columns. For example, DAQ’s Duplin County Air Monitoring Study produced limited insight into community concerns around illness, odor, and well-being, especially for people living very near swine facilities and manure-sprayed fields.^105^ Additionally, ongoing measurements by the National Atmospheric Deposition Program Ammonia Monitoring Network of NH_3_ and particle-phase ammonium in Sampson County are two week-integrated observations, using passive diffusion samplers returned to the laboratory for quantification by flow injection analysis, which have a documented 40% low bias compared to annular denuders.^122^ As a consequence, high NH_3_ events can go undetected: if NH_3_ exceeds the ATSDR acute standard for 8 h in 2 weeks, assuming 10 ppb NH_3_ at all other times, the reported NH_3_ would be only 30 ppb.

Eastern North Carolina residents are largely unprotected from CAFO-related air pollution by environmental regulation at all levels. In 2018, the North Carolina Legislature passed legislation^123^ to prevent further judicial action in favor of residents with nuisance claims.^124−126^ Now lawsuits are restricted to those living within 0.8 km of a facility, with residents who are experiencing ongoing issues of longer than one year having no recourse in the courts.^125^ At the same time, IASI ΔNH_3_ columns demonstrate that NH_3_ is enhanced further downfield than 0.8 km, particularly under calm and/or hot conditions, with NH_3_ exposures and inequalities driven in part by temperature-dependent NH_3_ volatization away from the source, and that CAFO-related air quality impacts are ongoing and unaddressed, at least since 2008. While the NCDEQ has developed some environmental justice-relevant initiatives because of the Settlement Agreement, including creating a fulltime Title VI Coordinator position, the design and implementation of the Duplin County Air Monitoring Study reflect an agency focused on compliance rather than residents’ concerns. A small step would be for NCDEQ to include oversampled, block group-scale IASI NH_3_ columns on the Community Mapping Tool to incorporate region-wide evidence of CAFO-related air pollution impacts into decision making. While the tool has the functionality to map NCDEQ AFO and NPDES permits, information on the locations of cesspits and manure irrigation fields that are not currently publicly available are relevant for understanding the distribution of CAFO emissions. There are no federal air quality policies for industrialized animal industries that would protect residents in absence of local and state-level action, both in North Carolina and other states. Over two decades ago, the EPA concluded there were insufficient data to determine which CAFOs required air permits. In 2005, the EPA made a deal with operators, who paid a small fine to fund an EPA study of their emissions, including NH_3_, in exchange for immunity from past and future enforcement actions until the EPA developed an emissions model and permit system that remain unfinished today. Emissions controls to address systematic NH_3_ inequalities are needed that, at a minimum, respond to embodied, longstanding, and community-scale concerns around swine CAFOs, with IASI poised to monitor the success of such policies should they develop.

## Supporting Information Available

Histograms of morning surface wind speeds and air temperatures, time windows of analysis, sample relative and absolute inequalities for determining background column density and ΔNH_3_ columns (April–August 2016–2021), calculation workflows, population weighting equation, block groups with populations of Black and African Americans, Hispanics and Latinos, American Indians, and non-Hispanic/Latinos whites equal to or greater than the mean across Eastern North Carolina, relative and absolute inequalities based on the 2020 decennial census and 2016–2020 ACS, ASOS station locations, monthly ΔNH_3_ columns, signal-to-noise ratio determination, comparison of MetOp-A and B population-weighted ΔNH_3_ columns, trends in population-weighted ΔNH_3_ and NH_3_ columns using constant demographic information, DAQ NH_3_ monitor locations, available ambient NH_3_ standards, description of computing inequalities in Potentially Underserved block groups, and NCDEQ Potentially Underserved block groups map (PDF)
