# Supplementary material for: Satellite Observations of Atmospheric Ammonia Inequalities Associated with Industrialized Swine Facilities in Eastern North Carolina
Source: Environ Sci Technol. 2025 Jan 29;59(5):2651–64. doi: 10.1021/acs.est.4c11922 (PMC11823455; doi:10.1021/acs.est.4c11922)
Supplement: Supplementary file 1 — es4c11922_si_001.pdf [file es4c11922_si_001.pdf]

Supporting Information

Satellite observations of atmospheric ammonia inequalities associated with industrialized swine facilities in Eastern North Carolina

AUTHOR NAMES: Akirah Epps[†,1], Isabella M. Dressel[†,1], Xuehui Guo[1], Maghogho Odanibe[1], Kimberly P. Fields[2], Ann Marie G. Carlton[3], Kang Sun[4,5], Sally E. Pusede[1]*

*Corresponding author: sepusede@virginia.edu

AUTHOR ADDRESSES:

[1]Department of Environmental Sciences, University of Virginia, Charlottesville, Virginia 22904, United States

[2]Carter G. Woodson Institute for African American and African Studies, University of Virginia, Charlottesville, Virginia 22904, United States

[3]Department of Chemistry, University of California Irvine, Irvine, California, 92697, United States

[4]Department of Civil, Structural and Environmental Engineering, University at Buffalo, Buffalo, New York 14260, United States

[5]Research and Education in eNergy, Environment and Water (RENEW) Institute, University at Buffalo, Buffalo, New York 14260, United States

[†]A. E. and I. M. D. contributed equally to this article.

SI includes: 18 pages, 8 figures, 5 tables, 1 equation, and 2 appendices

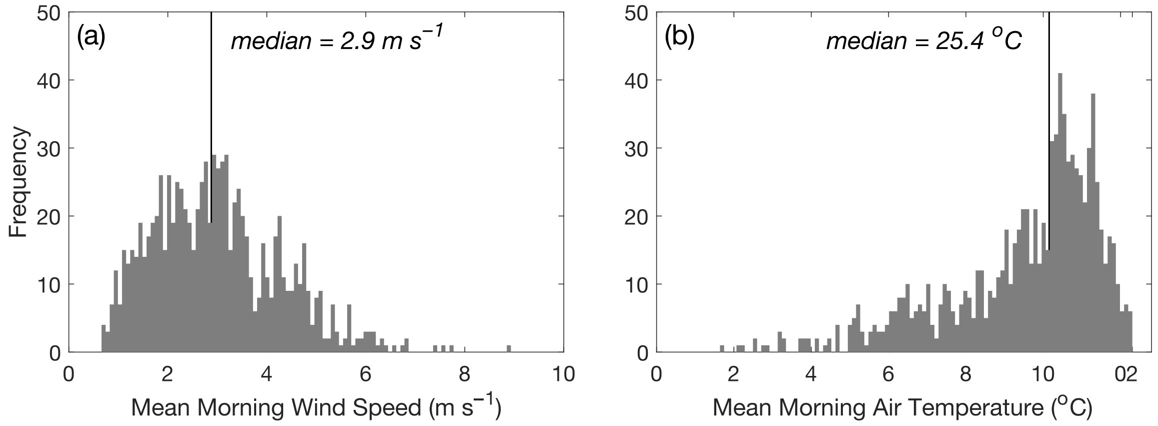

Figure S1. Histograms of morning (8 am–12 pm local time, LT) surface wind speeds (a) and air temperatures (b) over April–August 2016–2021 with medians displayed.

Table S1. Time windows of analysis.

Satellite	Column Averaging Period	Corresponding Background Period
MetOp-A	April–August 2016–2021	April–August 2016–2021
MetOp-A	Calm days: April–August 2016–2021, days with mean morning surface wind speeds below the median	April–August 2016–2021
MetOp-A	Windy days: April–August 2016–2021, days with mean morning surface wind speeds above the median	April–August 2016–2021
MetOp-A	Cool days: April–August 2016–2021, days with mean morning surface air temperatures below the median	April–August 2016–2021
MetOp-A	Hot days: April–August 2016–2021, days with mean morning surface air temperatures above the median	April–August 2016–2021
MetOp-A	April–August 2018–2021	April–August 2018–2021
MetOp-A	April–August 2014–2017	April–August 2014–2017
MetOp-A	April–August 2011–2013	April–August 2011–2013
MetOp-A	April–August 2008–2010	April–August 2008–2010
MetOp-B	April–August 2022–2023	April–August 2022–2023
MetOp-B	April–August 2018–2021	April–August 2018–2021
MetOp-B	April–August 2014–2017	April–August 2014–2017

Table S2. Relative and absolute NH_3 inequalities for Black and African Americans, Hispanics and Latinos, and American Indians based on ΔNH_3 columns computed using a tropospheric NH_3 background of each decile threshold for April–August 2016–2021. The percent population of Black and African Americans, Hispanics and Latinos, American Indians, and non-Hispanic/Latino whites in block groups with ΔNH_3 at each decile threshold is also shown.

		**Relative Inequalities (%)**			**Absolute Inequalities ($\times 10^{14}$ molecules cm^{-2})**			**Population in Block Groups with ΔNH_3 Columns (%)**			
**Percentile**	**Background NH_3 ($\times 10^{15}$ molecules cm^{-2})**	**Black/African Americans**	**Hispanics/ Latinos**	**American Indians**	**Black/African Americans**	**Hispanics/ Latinos**	**American Indians**	**Black/African Americans**	**Hispanics/ Latinos**	**American Indians**	**Non-Hispanic/ Latino Whites**
0	0	11 ± 1	14 ± 1	20 ± 1	6.5 ± 0.6	8.6 ± 0.8	12.9 ± 0.9	25%	10%	2%	56%
10th	3.8	27 ± 3	35 ± 3	49 ± 3	6.3 ± 0.6	8.4 ± 0.8	12.7 ± 0.8	26%	10%	2%	56%
20th	4.4	31 ± 4	41 ± 5	57 ± 4	5.3 ± 0.6	7.6 ± 0.8	11.7 ± 0.8	26%	10%	3%	55%
30th	4.7	30 ± 5	42 ± 5	61 ± 5	4.2 ± 0.6	6.6 ± 0.8	10.6 ± 0.8	27%	10%	3%	54%
40th	5.0	23 ± 5	40 ± 6	58 ± 5	2.9 ± 0.7	5.5 ± 0.8	8.9 ± 0.8	28%	10%	3%	53%
50th	5.4	5 ± 6	36 ± 7	41 ± 6	0.6 ± 0.7	4.9 ± 0.9	5.6 ± 0.8	29%	10%	3%	51%
60th	5.7	2 ± 7	54 ± 8	30 ± 7	0.3 ± 0.8	7.4 ± 1.1	3.6 ± 0.8	30%	10%	4%	51%
70th	6.0	5 ± 8	66 ± 9	6 ± 8	0.5 ± 0.9	9.9 ± 1.2	0.7 ± 0.8	29%	10%	4%	51%
80th	6.4	13 ± 9	71 ± 9	–41 ± 11	1.5 ± 1.1	11.9 ± 1.4	–3.7 ± 1.0	28%	10%	6%	51%

SI Appendix 1: ΔNH_3 columns

NH_3 column enhancements (ΔNH_3 columns) were computed as NH_3 columns above and then less, an observationally-determined tropospheric NH_3 background column density value. The tropospheric NH_3 background for a given multiyear period was identified as the highest decile of statewide oversampled NH_3 columns that, after subtracting that value from the NH_3 columns, gave statistically equivalent absolute block group-scale inequalities in ΔNH_3 and NH_3 columns based on the standard mean error in Eastern North Carolina. The tenth percentile was identified using NH_3 columns in April–August 2016–2021 (Table S2). A workflow of the process to identify the tenth percentile as the tropospheric NH_3 background follows:

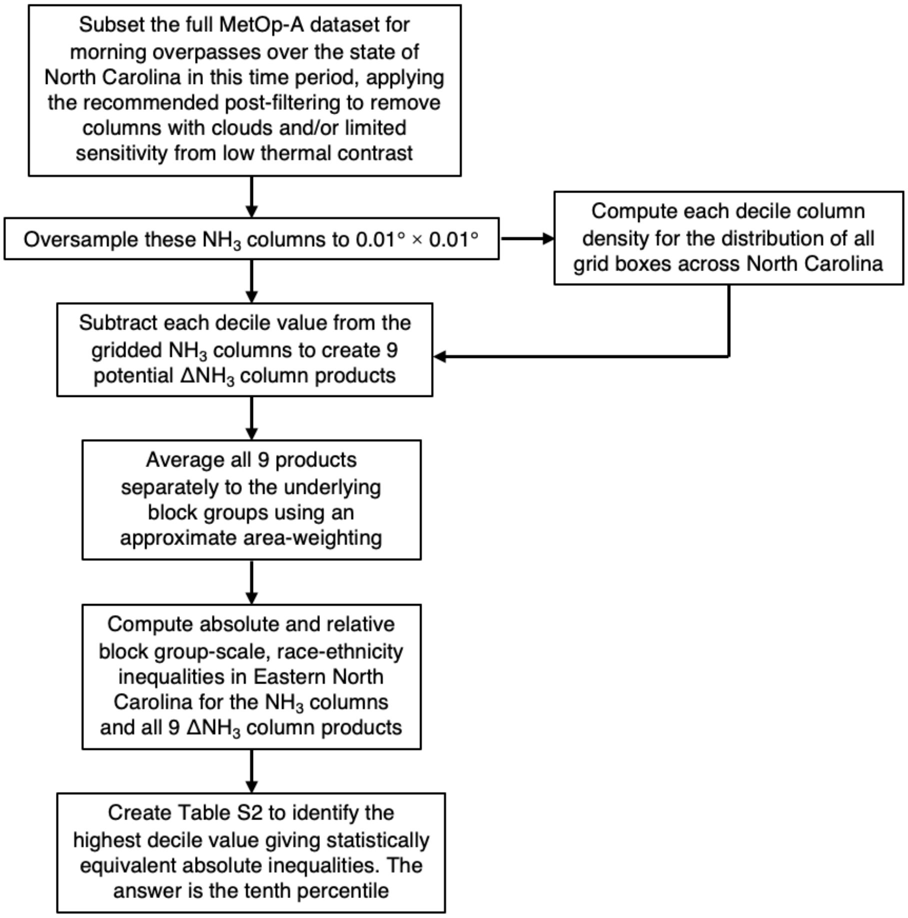

ΔNH_3 columns in each period use a unique, corresponding tropospheric NH_3 background column density (Table S1). That background value is always equal to the tenth percentile of the distribution across North Carolina for those years. A workflow for computing ΔNH_3 columns follows, with ΔNH_3 columns in April–August 2018–2021 from MetOp-A used as an example:

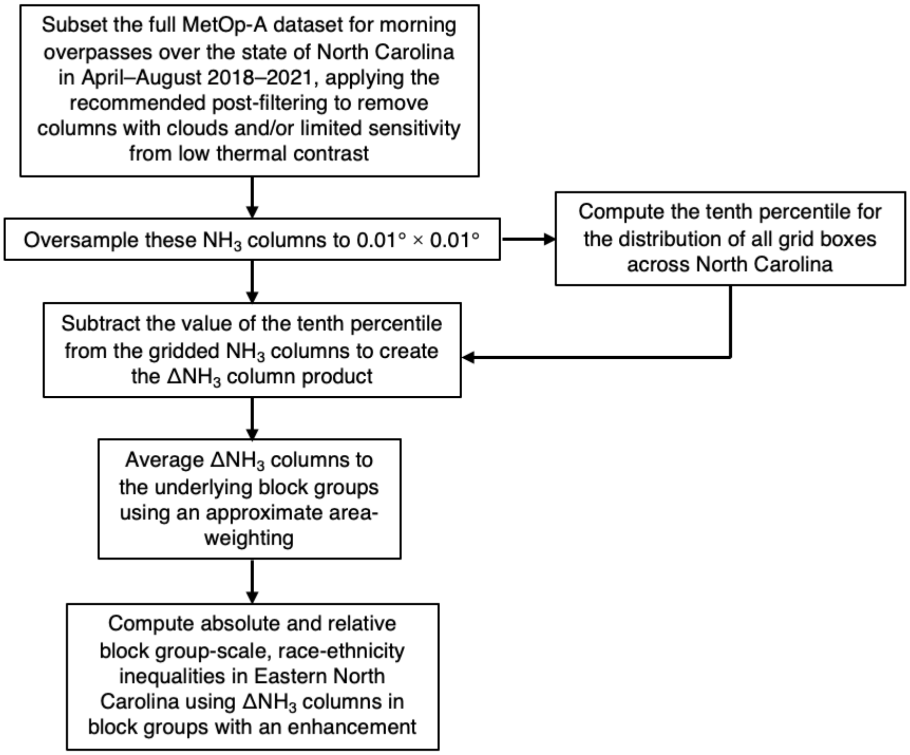

Equation S1. ΔNH_3 columns are population weighted as equal to the product of the block group-averaged ΔNH_3 columns ($\Delta NH_{3,j}$) and race-ethnicity group population (p_j) in each block group summed over all block groups included in the calculation (*n*). The summation is divided by the race-ethnicity group population (p_j) summed over all block groups included in the calculation.

(Eq. S1) Population-weighted $\Delta NH_{3,j} = \sum^n \Delta NH_{3,i}\, p_{i,j} / \sum^n p_{i,j}$

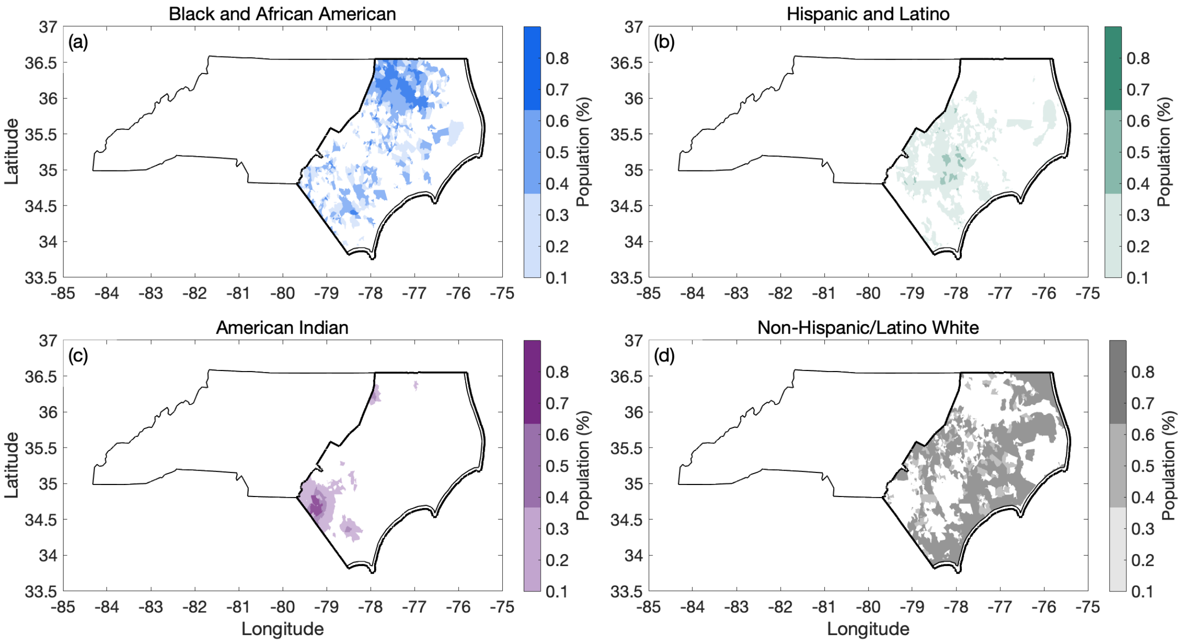

Figure S2. Inequalities are based on block groups with populations of that group equal to or greater than the mean across Eastern North Carolina counties for Black and African Americans (a), Hispanics and Latinos (b), American Indians (c), and non-Hispanic/Latinos whites (d).

Table S3. Relative and absolute block group-scale NH_3 inequalities in April–August 2016–2020 based on the 2020 decennial census and the 2016–2020 American Community Survey (ACS).

	Relative Inequality (%)			Absolute Inequality ($\times 10^{14}$ molecules cm^{-2})		
	**Black/African Americans**	**Hispanics/ Latinos**	**American Indians**	**Black/African Americans**	**Hispanics/ Latinos**	**American Indians**
2020 Decennial Census	27 ± 3	35 ± 3	48 ± 3	6.2 ± 0.6	8.3 ± 0.8	12.6 ± 0.8
5-Year 2016–2020 ACS with mean populations from the ACS	23 ± 3	34 ± 3	44 ± 3	5.3 ± 0.6	8.4 ± 0.8	11.4 ± 0.9
5-Year 2016–2020 ACS with mean populations from the 2020 Decennial Census	22 ± 3	34 ± 3	43 ± 3	5.1 ± 0.6	8.4 ± 0.8	11.2 ± 0.8

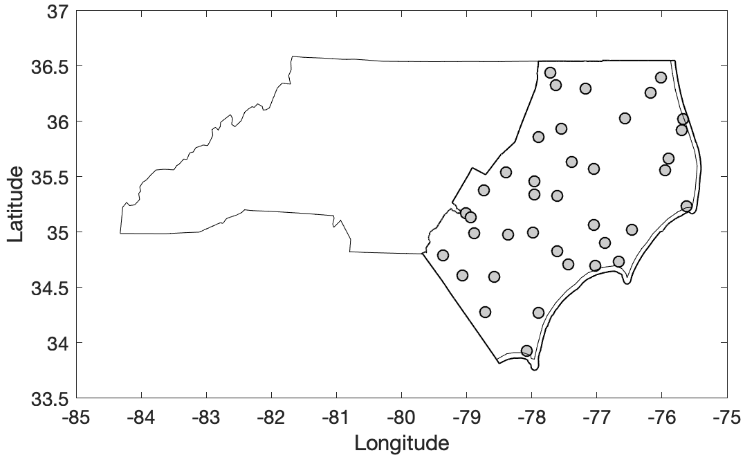

Figure S3. Surface monitor locations of meteorological measurements from the Automated Surface Observing System and Automated Weather Observing System over 2008–2021. Observations may not be available in each year.

Figure S4. Monthly IASI NH_3 columns measured during the morning overpass in 2016–2021 oversampled to $0.01^o \times 0.01^o$.

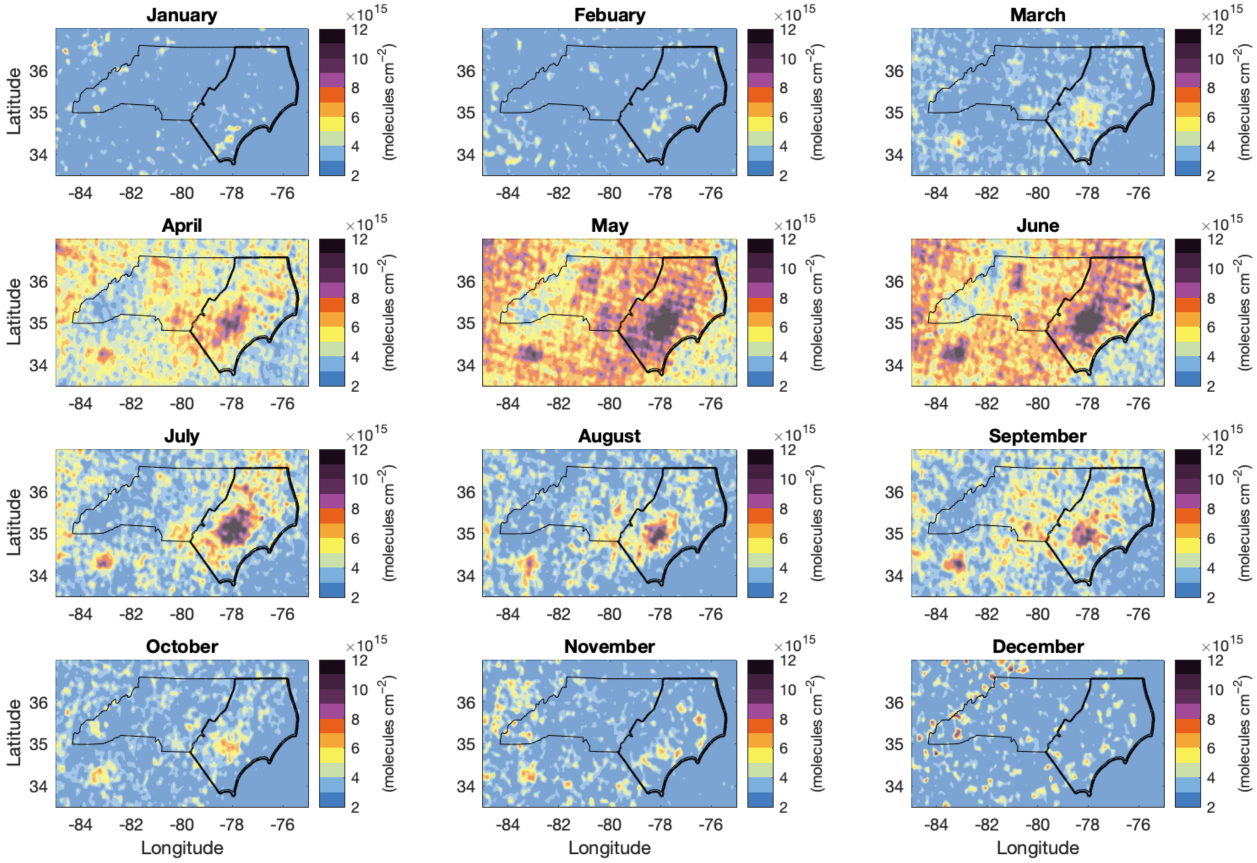

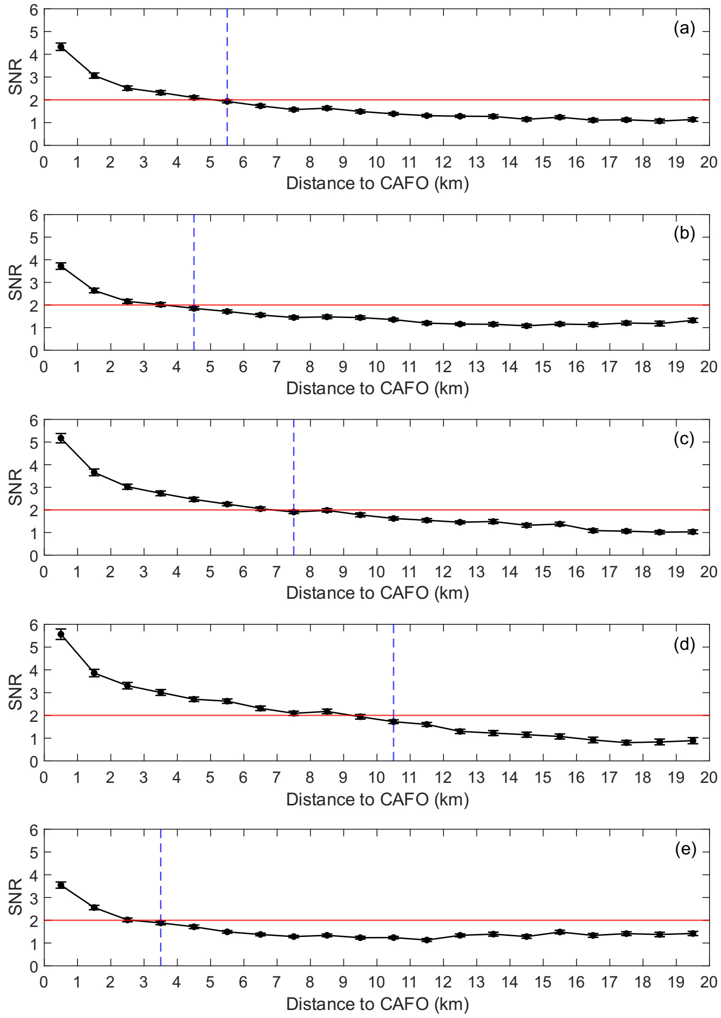

Figure S5. Signal to noise ratio (SNR or *S*/*N*) versus distance the nearest permitted swine facility in April–August 2016–2021 on all days (a) and on days with: mean morning (8 am–12 pm LT) wind speeds above the median wind speed (b), mean morning wind speeds below the median wind speed (c), mean morning air temperatures above the median air temperature (d), and mean morning air temperatures below the median air temperature (e). The signal (*S*) is defined as the mean of ΔNH_3 columns at each distance. The noise (*N*) is defined as the mean of two standard deviations of the ΔNH_3 columns (*S*) at 15–20 km away from the nearest permitted swine facility on all days. The blue line in each panel is the ΔNH_3 value where *S/N* equals 2.

Table S4. Population-weighted ΔNH_3 columns and relative and absolute inequalities in ΔNH_3 columns from MetOp-A and B in 2014–2017 and 2018–2021.

	Population-weighted ΔNH_3 columns ($\times 10^{14}$ molecules cm^{-2})				Relative Inequality (%)			Absolute Inequality ($\times 10^{14}$ molecules cm^{-2})		
	**Whites**	**Black and African Americans**	**Hispanics and Latinos**	**American Indians**	**Black and African Americans**	**Hispanics and Latinos**	**American Indians**	**Black and African Americans**	**Hispanics and Latinos**	**American Indians**
**2014–2017**										
MetOp-A	18.0 ± 0.4	22.2 ± 0.5	25.0 ± 0.7	29.6 ± 0.7	21 ± 3	33 ± 4	49 ± 4	4.2 ± 0.6	7.0 ± 0.8	11.6 ± 0.8
MetOp-B	19.9 ± 0.4	24.8 ± 0.5	27.6 ± 0.7	32.8 ± 0.8	22 ± 3	32 ± 4	49 ± 4	4.9 ± 0.7	7.7 ± 0.8	12.9 ± 0.9
**2018–2021**										
MetOp-A	23.0 ± 0.5	29.7 ± 0.5	31.6 ± 0.7	38.5 ± 0.8	25 ± 3	31 ± 3	50 ± 3	6.6 ± 0.7	8.6 ± 0.9	15.5 ± 0.9
MetOp-B	28.6 ± 0.5	36.4 ± 0.6	40.5 ± 0.8	47.2 ± 1.0	24 ± 2	34 ± 3	49 ± 3	7.7 ± 0.8	11.9 ± 1.0	18.5 ± 1.1

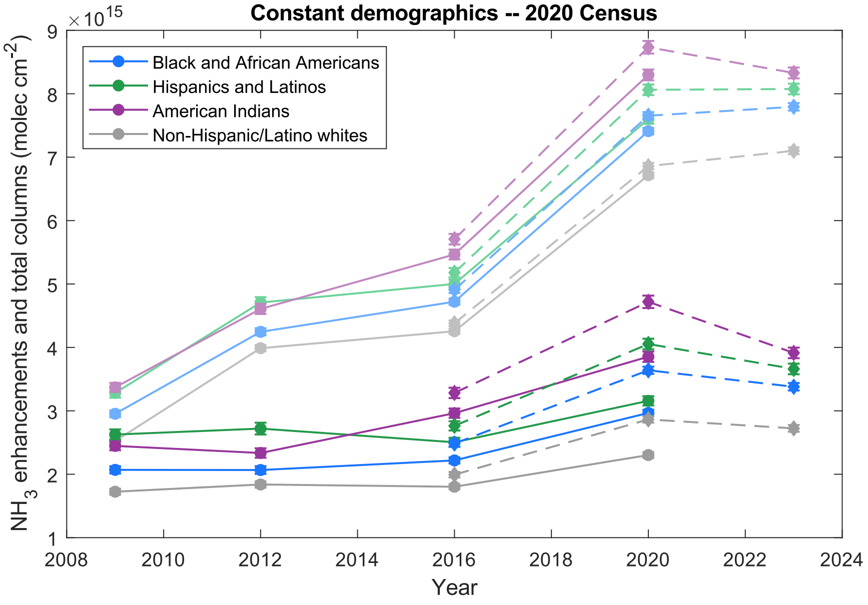

Figure S6. Population-weighted, block group-scale NH_3 (pastel) and ΔNH_3 columns (bright) in Eastern North Carolina for Black and African Americans (blue), Hispanics and Latinos (green), American Indians (purple), and non-Hispanic/Latino whites (gray). IASI observations from MetOp-A (circles, solid line) in April–August in 2008–2010, 2011–2013, 2014–2017, and 2018–2021 and MetOp-B (diamonds, dashed line) in April–August in 2014–2017, 2018–2021, and 2022–2023. Standard mean errors are similarly sized as the markers and omitted for clarity.

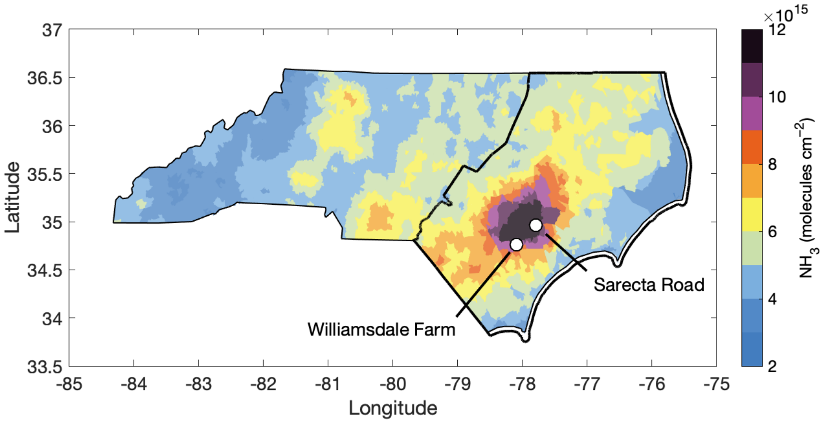

Figure S7. DAQ NH_3 monitor locations at Williamsdale Farm in Wallace and Sarecta Road in Beulaville, North Carolina during the Duplin County air quality study, required as part of the 2018 Settlement Agreement, with the NH_3 columns over April–August 2016–2021 (Figure 1).

Table S5. Available ambient NH_3 standards.

Standard	Value	Exposure	Type	Notes
NAAQS	–	–	–	–
NC AAL	3.868 ppm	acute	emissions	must be met at the facility boundary
ATSDR	1.7 ppm	acute	mixing ratio	–
ATSDR	0.1 ppm	chronic	mixing ratio	–

SI Appendix 2. Computing inequalities in Potentially Underserved block groups.

The NC DEQ defines Potentially Underserved block groups across North Carolina (Figure S8) based on 2019 block group boundaries. We use block groups flagged as Potentially Underserved compared to the county, compared to the state, or compared to the county and state. We match 2019 Potentially Underserved block groups to 2020 block group boundaries based on the 2019 block group center point. The 2020 block groups are assigned as Potentially Underserved if their boundary contains the center point of a 2019 Potentially Underserved block group. This provides a total of 1,399 block group matches between 2019 and 2020 out of 1,413 total 2019 Potentially Underserved block groups. We compute inequalities as population-weighted block group-averaged differences in ΔNH_3 for total populations in Potentially Underserved block groups versus total populations in all other block groups in Eastern North Carolina.

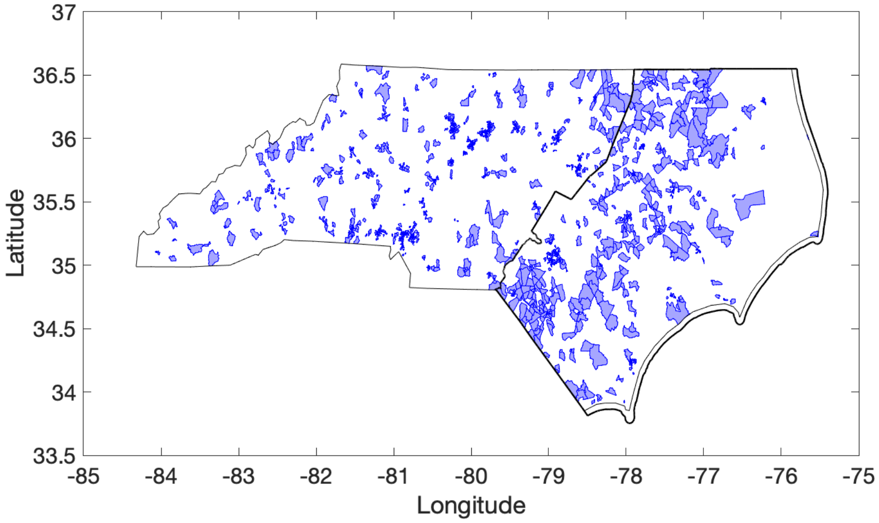

Figure S8. Potentially Underserved block groups across Eastern North Carolina defined using 2020 block group boundaries. Block groups are flagged as Potentially Underserved compared to the county, state, or county and state.
